# Paving the way for universal family planning coverage in Ethiopia: an analysis of wealth related inequality

**DOI:** 10.1186/s12939-015-0214-7

**Published:** 2015-09-14

**Authors:** Muluneh Yigzaw, David Zakus, Yehualashet Tadesse, Muluked Desalegn, Mesganaw Fantahun

**Affiliations:** Ethiopian Public Health Associetion, Addis Ababa, Ethiopia; Faculty of Community Services, School of Occupational and Public Health, Ryerson University, Toronto, Canada; Columbia University – ICAP in Ethiopia, Addis Ababa, Ethiopia; Amref Health Africa in Ethiopia, Addis Ababa, Ethiopia; School of Public Health, Addis Ababa University, Addis Ababa, Ethiopia

## Abstract

**Background:**

Family planning plays a significant role in reducing maternal and child mortality and ultimately in achieving national and international development goals. It also has an important role in reducing new pediatric HIV infections by preventing unwanted pregnancies among HIV positive women. Investing in family planning is one of the smart investments for development as population dynamics have a fundamental influence on the pillars of sustainable development, including that of a sustainable environment.

**Objective:**

To identify and quantify wealth related differences in family planning use between poor and rich Ethiopian women based on the Demographic and Health Survey asset based wealth quintiles.

**Methods:**

The proportion of women who used contraceptives during implementation of the 2011 and 2005 Ethiopia Demographic and Health Surveys was calculated across wealth quintiles. Data were stratified for place of residence to analyze and determine inequalities in family planning use separately for rural and urban women. Socioeconomic inequalities according to wealth were measured using the slope index of inequality and the relative index of inequality.

**Result:**

The absolute difference of contraceptive prevalence between poorest and richest women was over 25.3 percentage points (95 % CI = 18.9-31.7) in 2011. Contraceptive use was more than twice (RII: 2.6, 95 % CI = 2.0 - 3.3) as prevalent among the richest compared with the poorest.

**Conclusion:**

Despite efforts to provide contraceptives for free at all public health facilities, wealth based inequalities still prevail in Ethiopia. People at lower socioeconomic strata should be empowered more to avoid the root causes of inequality and to achieve national Health Sector Development Program Goals.

## Introduction

The causes of health outcomes are very complex and highly dictated by social determinants of health. Those at lower social strata are with the worst health outcomes [1], exacerbated by lower coverage of basic health and social services.

Since its endorsment in 2005 by the World Health Assembly, a growing number of countries are showing a comitment to address the bottle necks of universal access and Universal Health Coverage (UHC). Neverthless, given increased resource constraints and service complexity, the poorer countries need to focus on priority services and gradually expand to less priority services instead of advancing all costly services at one point in time [2]. Family planning is one of the priority and highly cost effective services in least developed nations with significant impact on population health and economic development [3, 4].

Family planning plays a significant role to reduce maternal and child mortality and ultimatlely to achieve national and international development goals including the Millennium Development Goals (MDGs), formulated in 2000 but expiring this year 2015. By reducing high-risk and unwanted pregnancies, family planning can significantly reduce the risk of maternal and child deaths [5–8]. Children born with short birth intervals or from a teenage mother are more likely to be stunted or underweight in later life [9, 10]. Conversely, malnutrition is closely linked to the major causes of under-five deaths in the developing world [11].

Apart from its effect on maternal and child mortality and morbidity, contraceptives have played a pivotal role in reducing new pediatrics HIV infections [12]. The World Health Organization (WHO) has identified family planning as one of the strategies for the prevention of mother-to-child transmission (PMTCT) of HIV [13]. It is proven that voluntary contraceptive use prevents many infants from HIV infections as compared to common interventions of treating the mother and the infant with anti-retroviral therapy [14–17]. Moreover, family planning as an HIV prevention approach is cost effective compared with other PMTCT approaches [14].

Investing in family planning is one of the smart investments for development as population dynamics have a fundamental influence on the pillars of sustainable development [18]. It is a balance and check for economic development and overall population growth and increases the capability of developing countries to provide all citizens with the required services including priority health services and adequate education. If high fertility rates continue to flourish among the poor, poverty will create a vicious circle as poor women and their families lose the opportunity to move successfully through education and into productive employment among others, including contributing to a more sustainable environment and reductions in pollution of all forms.

The last decade has witnessed a wide spread increase in the number of women wanting to avoid pregnancy and thus needing effective contraception in the developing world. However, the contraceptive prevalence rate remains unacceptably low in the majority of these nations [19] with a wide gap between the poor and the better off [20–22]. There were some 222 million women in developing countries wanting to plan their births but without having access to contraception in 2012 [23]. Despite the international consensus that access to safe and effective methods of contraception is a fundamental human right [24], women from poorest wealth quintiles have less likely to meet their needs for modern family planning methods [21, 22]. The unmet need for modern contraception is as high as 60 % in Sub-Saharan Africa, ranging from 81 % in Middle Africa to 17 % in Southern Africa [23].

Ethiopia showed substantial increase in the use of contraceptives in the last decade through a significant effort to scale up family planning services. The contraceptive prevalence rate increased from 8 % in 2000 to 28.6 % in 2011 among married women of reproductive age [25, 26]. However, about 26 % of women have not yet taken any type of contraceptive despite their demand. This high unmet need in contraceptives is probably because of a series of socio economic factors that affect the use of a range of health services including contraceptives.

The purpose of this study, therefore, was to identify and quantify wealth related differences in family planning use between poor and rich Ethiopian women based on the Demographic and Health Survey (DHS) asset based wealth quintiles. Furthermore we wanted to determine whether supplying contraceptives for free could address wealth related inequalities and to show that the national family planning coverage gap is a result of wealth related differences in family planning use and need satisfied.

## Methods

### Data sources

The authors analyzed the data of the 2011 Ethiopian Demographic and Health Survey (EDHS) and to some extent those of the 2005 EDHS to examine trends of inequality over five years. The study population was women of the reproductive age (15–49 years). Women were asked to respond to a series of questions related to their socio-demographic characteristics, reproductive behavior, use of maternal health services and the need for contraception. The detailed sampling procedure for the survey has been reported elsewhere [25].

### Variables

To measure wealth related inequality in family planning use, two outcome variables were used:

- Current contraceptive use: Extracted from the question “Are you currently doing something or using any method to delay or avoid getting pregnant?” The answer was dichotomized into: ‘Yes’ and ‘No’. When a woman also used a traditional method, she was put into the first category.
- Contraceptive needs satisfied: This is only for women who do not want to become pregnant and using contraception to avoid pregnancy. It is generated from the response to a number of questions in the women’s questionnaire.

The main independent variable analyzed in this study was the wealth index. It is a composite measure of a household's cumulative living standard (economic position). Information on the wealth index is based on data collected in the household questionnaire; including questions related to: the household’s ownership of consumer items; dwelling characteristics; drinking water sources; toilet facilities; and other characteristics such as number of people per room, bank account ownership, domestic servant in household, and ownership of agricultural land.

Wealth scores were much higher for urban women compared to rural women and resulted in more women in the richest quintile in urban areas and many more women in the poorest quintile in rural areas. To avoid this and to compare inequalities in contraceptive use and needs satisfied separately for rural and urban women, we used the national-level wealth scores as they were generated, and then divided each into quintiles separately in urban and rural areas. The detail methodology for wealth indexing is indicated elsewhere [27].

### Data analysis

Univariate and multivariate statistical analyses were employed to analyze the data. Contraceptive prevalence rate and contraceptive needs satisfied were analyzed across wealth quintiles in a weighted sample of 10287 and 9066 women from the 2011 and 2005 EDHS respectively. Wealth related inequalities were measured using the absolute index of inequality (SII) and the relative index of inequality (RII). This was mainly to measure and compare changes in absolute and relative wealth related inequalities over the five years between the two surveys [28–30]. Additionally, population attributable risk (PAR) and the population attributable risk percentage was used to quantify the contribution of wealth based inequalities to the national coverage gaps [31, 32].

To calculate SII and RII, individuals were cumulatively ranked according to ascending economic status. The exposure variable can thus be interpreted as a continuous measure, with a value of zero equivalent to the bottom economic distribution and a value of one equivalent to the top. SII was thus the prevalence rate difference and RII was the prevalence rate ratio between the highest and the lowest level of wealth.

Adjusted population attributable risks and attributable fractions were calculated to determine coverage gaps due to wealth. STATA’s Margins command was used to calculate population attributable risk and population attributable risk percent.

Data were adjusted for educational status of women, as well as other confounding factors, including: age of women; place of residence; number of living children; working status; and husband's desire for more children. Both crude and adjusted results were presented. All the analyses were carried out using STATA® 12(STATA Corp. Texas, USA).

## Results

A total of 10287 weighted number of married women from EDHS 2011 and 9066 women from DHS 2005 was included for the analysis. The mean age of the study participants was 30.7 ranging from 15 to 49 years. Study participants background characteristics are presented in Table 1.

**Table 1:** Frequency distribution of married women according to background characteristics, Ethiopia Demographic and Health survey (EDHS 2005 and 2011)

Variables	2011	2005
	N (%)	N (%)
Age		
15–18	528 (5.1)	533 (5.9)
19–25	2768 (26.9)	2492 (27.5)
26–35	4088 (39.7)	3449 (38.1)
36–49	2903 (28.2)	2592 (28.6)
Total	10287	9066
Place of residence		
Urban	1843 (17.92)	959 (10.6)
Rural	8444 (82.08)	8107 (89.4)
Total	10287	9066
Religion		
Ethiopian Orthodox	4490 (43.69)	4132 (46.1)
Muslim	3187 (31.01)	2903 (32.4)
Protestant	2432 (23.66)	1804 (20.1)
Traditional	169 (1.64)	134 (1.5)
Total	10287	8973
Educational Level		
No education	6735 (65.47)	7094 (78.2)
Primary	2862 (27.82)	1402 (15.5)
Secondary	377 (3.67)	485 (5.4)
Higher	312.8 (3.04)	85 (0.9)
Total	10287	9066
Wealth index		
Q1	2077 (20.19)	1759 (19.4)
Q2	2117 (20.58)	1892 (20.9)
Q3	2083 (20.25)	1903 (21.0)
Q4	1923 (18.69)	1823 (20.1)
Q5	20287 (20.29)	1689 (18.6)
Total	10287	9066
Number of living children		
No living children	1018 (9.9)	800 (8.8)
1–2 living children	3193 (31.0)	2628 (29.0)
≥ 3	6076 (59.1)	5638 (62.2)
Total	10287	9066
Number of people in the household		
≤ 4	3337 (32.4)	2681 (29.6)
5–7 people	4910 (47.7)	4470 (49.3)
≥ 8	2040 (19.8)	1915 (21.1)
Total	10287	9066
Respondent currently working		
Yes	3678 (35.8)	2244 (24.7)
No	6600 (64.2)	6822 (75.3)
Total	10277	9066
Husband’s desire for more children		
Husband wants more	2504 (24.5)	1540 (17.1)
Wants same	4234 (41.4)	2981 (33.0)
Wants fewer	887 (8.7)	433 (4.8)
Unknown	2598 (25.41)	4077 (45.2)
Total	10223	9031
Contraceptive needs satisfied		
Yes	2944 (52.11)	1334 (29.0)
No	2705 (47.9)	3272 (71.0)
Total	5949	4606
Contraceptive use		
Yes	2944 (28.61)	1334 (14.7)
No	7344 (71.39)	7732 (85.3)
Total	10288	9066

Table 2 shows the average coverage, SII and RII in contraceptive use and needs satisfied among Ethiopian women. The 2011 EDHS showed that the contraceptive prevalence rate (CPR) ranged from 51.8 % among the richest to 13.3 % among the poorest and the difference was significant (*p* < 0.001). Contraceptive usage between the poorest and the richest women differed by 25 percentage points in favor of the richest and, the contraceptive prevalence rate among the richest was more than two fold higher than the poorest. The national contraceptive needs satisfied were as high as 77 and 29 % among women from richest and poorest households, respectively. There was a significant difference in the contraceptive needs satisfied between richest and poorest women with the richest being 30 percentage points higher than the poorest.

**Table 2:** Average prevalence/coverage, absolute and relative wealth related inequalities in family planning use and contraceptive needs satisfied among Ethiopian women, Ethiopia 2005 and 2011

EDHS 2011						EDHS 2005				
Variable	%	Crude SII	Adj SII	Crude RII	Adj RII	%	Crude SII	Adj SII	Crude RII	Adj RII
		(95 % CI)	(95 % CI)	(95 % CI)	(95 % CI)		(95 % CI)	(95 % CI)	(95 % CI)	(95 % CI)
Contraceptive needs satisfied										
Q1	29.5					10.4				
Q2	44.6					14.5				
Q3	45.6	51.5	30.3	3.11	1.85	23.5	54.6	31.9	10.38	3.40
Q4	53.0	(44.6 – 58.5)	(20.8 – 39.9)	(2.5 – 3.9)	(1.5 – 2.3)	28.3	(49.2–60.1)	(24.0 –39.7)	(7.1 – 15.1)	(2.4 –4.8)
Q5	77.5					58.5				
Contraceptive use										
Q1	13.3					4.2				
Q2	22.2					6.6				
Q3	24.4	42.6	25.3	4.96	2.55	12.0	38.7	21.7	17.00	5.13
Q4	31.7	(37.6 –47.5)	(18.9– 31.7)	(3.9 – 6.3)	(2.0 – 3.3)	15.5	(34.8–42.6)	(16.9–26.4)	(11.7 – 24.6)	(3.5– 7.5)
Q5	51.8					37.0				

Table 3 shows the average contraceptive coverage, and SII and RII for women living in rural areas. Thirteen percent of rural women in the poorest quintile and 35.7 % of women in the wealthiest quintile were using either modern or traditional methods of contraception in 2011.

**Table 3:** Average prevalence/coverage, absolute and relative wealth related inequalities in family planning use and contraceptive needs satisfied among rural women, Ethiopia 2005 and 2011

EDHS 2011						EDHS 2005				
Variable	%	Crude SII	Adj SII	Crude RII	Adj RII	%	Crude SII	Adj SII	Crude RII	Adj RII
		(95 % CI)	(95 % CI)	(95 % CI)	(95 % CI)		(95 % CI)	(95 % CI)	(95 % CI)	(95 % CI)
Contraceptive needs satisfied										
Q1	30.1					9.4				
Q2	39.5					13.6				
Q3	43.3	32.0	23.6	2.1	1.7	21.1	32.3	26.5	5.0	3.7
Q4	49.1	(22.9–41.0)	(14.5–32.7)	(1.7–2.7)	(1.4–2.2)	26.8	(25.9 –38.7)	(20.1–32.0)	(3.5 –7.1)	(2.6 – 5.2)
Q5	57.7					35.3				
Contraceptive use										
Q1	13.3					3.7				
Q2	19.1					6.1				
Q3	23.0	25.8	18.6	3.1	2.3	10.8	21.0	16.2	7.3	4.8
Q4	25.9	(20.0–31.5)	(12.9–24.3)	(2.4–4.1)	(1.8–2.9)	14.3	(16.9–25.2)	(12.4–19.9)	(5.0–10.6)	(3.4–6.8)
Q5	35.7					19.9				

About 33 % and 52 % of women from poorest and richest households in urban areas, respectively, were using contraceptives in 2011. Fifty-five percent of the poorest and 81.6 % of the richest quintiles had contraceptive needs satisfied (SII = 25.6 percentage points, RII = 1.4) (Table 4). Significant difference was also observed in rural women with a lower absolute difference and a slight higher relative inequality (SII = 23.6 percentage points, RII = 1.7) (Table 3). The contraceptives needs satisfied followed a linear pattern of inequality among rural women while a bottom inequality pattern was observed in urban women with a coverage level of greater than 80 % among those women in the middle, richer and richest wealth quintiles.

**Table 4:** Average prevalence/coverage, absolute and relative wealth related inequalities in family planning use and contraceptive needs satisfied among urban women, Ethiopia 2005 and 2011

EDHS 2011						EDHS 2005				
Variable	%	Crude SII	Adj SII	Crude RII	Adj RII	%	Crude SII	Adj SII	Crude RII	Adj RII
		(95 % CI)	(95 % CI)	(95 % CI)	(95 % CI)		(95 % CI)	(95 % CI)	(95 % CI)	(95 % CI)
Contraceptive needs satisfied										
Q1	55.0					46.4				
Q2	75.9					66.8				
Q3	82.9	31.9	25.6	1.5	1.4	75.8	36.9	24.1	1.7	1.4
Q4	87.4	(19.1–44.6)	(10.3–40.9)	(1.3–1.9)	(1.1–1.8)	81.5	(19.9–53.8)	(6.2–42.1)	(1.3–2.4)	(1.1–1.9)
Q5	81.6					77.3				
Contraceptive use										
Q1	33.4					27.1				
Q2	53.4					47.6				
Q3	60.1	23.7	22.5	1.6	1.6	51.8	28.0	20.0	1.8	1.6
Q4	63.1	(11.2–36.2)	(9.1–35.9)	(1.2–2.0)	(1.2–2.0)	55.8	(18.0–37.9)	(8.7–31.3)	(1.4–2.4)	(1.2–2.0)
Q5	52.4					51.2				

The contraceptive needs satisfied among poorest rural women increased from 9 % in 2005 to 30 % in 2011. Moreover, the adjusted rate ratio for contraceptive needs satisfied among women living in rural Ethiopia dropped from 3.7 in 2005 to 1.7 in 2011 (Table 3). However, as the intervention coverage increases, the absolute inequality in the contraceptive needs satisfied between the poor and the better off was not significantly changed both in urban and rural areas between 2005 and 2011. The contraceptive prevalence rate ratio among rural women dropped from 4.8 to 2.3. But the rate difference increased from 16 percentage points in 2005 to 19 percentage points in 2011 (Table 3). For urban Ethiopia, neither the absolute nor the relative inequality showed a significant change (Table 4).

The national gap in the contraceptive needs satisfied was about 48 % in 2011(Table 5). Wealth contributed about 53 % of this coverage gap (PAR% = 53.0, 95 % CI = 44.0–60.6). In general, the coverage gap in the contraceptive needs satisfied was much lower in urban women than in rural women (23 vs 55). However, the population attributable risk did not significantly differ between urban and rural women (11.3 vs 12.2) (Table 5).

**Table 5:** Average gap in family planning use and contraceptive needs satisfied and within-country wealth based inequality in coverage gap, Ethiopia DHS 2011

Variable	Coverage gap (95 % CI)		PAR, percentage points (95 % CI)	PAR % (95 % CI)
	National	In richest quintile		
National				
Contraceptive needs satisfied	47.9 (45.1–50.7)	22.5 (18.6–27.0)	25.4 (21.1–29.5)	53.0 (44.0–60.6)
Contraceptive use	71.4 (69.4–73.3)	48.2 (44.3–52.2)	23.2 (19.3–26.9)	32.4 (27.0–(37.5)
Rural				
Contraceptive needs satisfied	55.0 (51.5–58.5)	42.9 (37.9–47.9)	12.2 (8.6–15.7)	22.1 (15.1–28.5)
Contraceptive use	76.6 (74.2–78.8)	65.4 (61.5–69.2)	11.2 (8.5–13.8)	14.6 (10.9–18.1)
Urban				
Contraceptive needs satisfied	34.1 (19.1–(27.0)	22.8 (7.8–16.8)	11.3 (7.11–15.4)	41.4 (14.8–59.7)
Contraceptive use	47.5 (44.0–51.1)	38.0 (32.1 (44.3)	9.5 (4.4–14.5)	20.0 (8.5–30.0)

## Discussion

Our study shows that despite a tremendous investment and an enormous increase in family planning use, significant disparity still exists between the poor and better off women in Ethiopia. Many studies conducted in the developing world have also showed that wealthier women are more likely to use family planning methods and maternal health care services than their less wealthy counterparts [33–35]. The relative inequality in family planning use and contraceptive needs satisfied between wealthiest and poorest women significantly dropped over five years period (between 2005 and 2011) in rural Ethiopia. However, such a trend was not observed among urban women. Significant change was also not observed in absolute wealth related inequality in both urban and rural areas. The coverage seems to have rapidly expanded to well-off groups leaving the worse off groups in urban areas unchecked which is regarded as an unacceptable trade off by the World Health Organization [2].

The Ethiopian Ministry of Health introduced the health extension program in 2002, deploying Health Extension Workers (HEWs) educated on 16 packages of health service including family planning in rural Ethiopia [36]. HEWs were working almost in all kebeles (the smallest administrative unit in Ethiopia, akin to an extended neighborhood) of the country in 2011 [37] during the time the 2011 EDHS was conducted. The contraceptive prevalence rate increased by almost three and half fold after introduction of the health extension program and the relative inequalities in family planning use between the poorest and wealthiest women also started to drop in rural Ethiopia in the period between 2005 and 2011. Nevertheless, the coverage for family planning among the poorest is thus far significantly lower than the coverage among the wealthiest groups and the absolute inequality has either worsened or not shown significant improvement.

On top of deploying HEWs to provide family planning and other essential services to the rural underserved populations, the ministry of health has supplied contraceptives free of charge at public health facilities. However, supplying contraceptives for free is only one step to achieve universal family planning coverage; poor quality services [38], frequent stock outs and other distal causes like legal and policy gaps can hinder family planning use especially among poor women. Wealthiest women may, for instance, use other service alternatives if the service quality deteriorates or if there are frequent stock outs at public facilities. The persistent wealth related gaps in family planning use and needs satisfied between 2005 and 2011 among urban women might be partly due to quality issues and stock outs at public facilities which leave the poor without access and alternatives as the private for profit facilities are unaffordable to them. Moreover, the poor usually have no voice in public decision-making, unless consulted through participatory methods, and are thereby excluded from a number of social and economic services.

Addressing the ‘cause of causes’ , which is literally known as structural determinants, for family planning and other health service use requires systematic and well-designed investigations based on local contexts [39]. Structural reforms related to policy and legal frameworks might be crucial to reach all women in need of family planning and even important to create demand among those who have minimal appetite for family planning.

Though the health system most often focuses on the immediate causes of ill health and service coverage, it also must play a central role in addressing the ‘cause of causes’. It needs to enable community participation [40, 41]; inter-sectorial collaboration and most importantly, help shape societal norms and values [42, 43]. Participation of the poor during the planning, design and monitoring of health programs results in high service utilization and service coverage [44, 45]. Inequality in family planning and other reproductive health services use is usually exacerbated if women are passive targets of health programs [46]. Full participation of the poor (including both women and men) at planning phase and during all stages of program implementation can give chance to family planning programmers to overcome bottlenecks to family planning use at all levels of facilities. It also boosts local resource mobilization and service sustainability. Re-defining and strengthening the primary health system is, therefore, crucial to address these problems of inequality.

Implementation research, too, can play a crucial role to make design changes to current strategies [47], to better reach the poor and to ensure that the health extension program has considered community participation as an important contributor in reaching all population groups regardless of their social position and in how to design effective and efficient strategies to increase the potential of community participation, which can so often be squandered [40].

Indirect costs might also be other obstacles for family planning utilization in rural areas where living depends on subsistence agriculture. Even though contraceptives are supplied free of charge to all women served at public health facilities, the poor may be excluded due to indirect costs such as opportunity losses and transportation costs to public facilities. Mobilizing local resources might fill this gap and facilitate service utilization by the poor for family planning and other priority services.

Gender inequality is more pronounced amongst poorest quintiles [48] clearly indicating that household assets are more likely to be controlled by men in the poorest households. Poorest women might not, therefore, get a green light to use contraceptives (or those of their choice) and hence be denied optimal family planning opportunities. Empowering those women at lower socioeconomic strata might, thus help to increase women’s decision making power at household level and thereby decrease the gap in contraceptive use between the poor and the better off.

Wealth related inequality accounted for 53 % (95 % CI = 44.0 – 60.6 %) of the coverage gap in the contraceptive needs satisfied in 2011; with the national coverage gap dropping to 22.5 % if no wealth related inequality existed. Hosseinpoor et al. reported that wealth related inequality accounted for 51 % of the coverage gap in Namibia, 47 % in Madagascar and 20 % in Chad based on demographic and health surveys conducted between 2000 and 2008 [33] which indicates that within country wealth related inequalities vary widely among countries as a cause of contraceptive use variation. However while within country inequalities differ among countries, its contribution to the national coverage gaps were significant in most Sub-Saharan Africa countries. It is, therefore, identified further as a major challenge in the effort to reach universal family planning coverage and must be seriously dealt with.

Our study has some limitations. First of all, we used cross-sectional data, which can only show associations rather than causal relationships between explanatory variables and the outcomes of interest, though two discrete points in time were used with the two DHSs. Second, wealth quintiles represent only relative wealth differences and the inequality observed in this study might be because of other forms of disparity (e.g. education). We, however, conducted multivariate and stratified analysis to control all these forms of disparities to come up with refined results. We analyzed, for instance, contraceptive use for rural and urban women separately to avoid disparities related to geographic access. Major variables considered as confounders such as education, age of women, place of residence, number of living children, working status and husband's desire for more children were included in the multivariate analysis model. However, we did not adjust for other factors that might influence contraceptive use among women but that were not part of the DHS (e.g. contraceptive stock-outs, health system empathy, etc.). Thus, our findings need to be interpreted with caution, since women may have used or may have been excluded from family planning services for reasons not included in the regression equations.

## Conclusion

Over all, the study result shows that wealth based inequalities in contraceptive use still prevail in Ethiopia despite the efforts to provide them for free at all public health facilities. Such existing wealth inequalities will, nonetheless, continue to pose a challenge to achieve national Health Sector Development Program targets and universal family planning coverage will not be realized. All actors in the arena have to understand that the cost of medical services is not the only reason for health disparity between the poor and the rich. Unless properly empowered to use the service, people at lower social stratum will be left behind and will be excluded from these (and other) priority services. The source of inequalities are deep rooted and multifactorial. The root causes of inequity and inequality should be systematically identified and targeted to make the services universal to all groups and achieve the national Health Sector Development Program goals. Apart from deep rooted structural causes, factors at the health system level should also be carefully scrutinized as quality of the service, hospitality factors and frequent stock outs might prevent the poor from using family planning services. Further qualitative and quantitative studies should also be carried out to find out the multi-level factors that exclude poorest women from family planning services apart from financial constraints, and to continue monitoring success in achieving universal health service goals.

## Abbreviations

UHC: Universal Health Coverage
MDGs: Millennium Development Goals
WHO: World Health Organization
PMTCT: Prevention of mother to child transmission of HIV
DHS: Demographic and Health Survey
EDHS: Ethiopia Demographic and Health Survey
RII: Relative index of inequality
SII: Slop index of inequality
CPR: Contraceptive prevalence rate
PAR: Population attributable risk
HEW: Heath extension worker
